# The Gly^16^ Allele of the G16R Single Nucleotide Polymorphism in the β_*2*_-Adrenergic Receptor Gene Augments the Glycemic Response to Adrenaline in Humans

**DOI:** 10.3389/fphys.2017.00661

**Published:** 2017-09-05

**Authors:** Kim Z. Rokamp, Jonatan M. Staalsø, Morten Zaar, Peter Rasmussen, Lonnie G. Petersen, Rikke V. Nielsen, Niels H. Secher, Niels V. Olsen, Henning B. Nielsen

**Affiliations:** ^1^Department of Anesthesia, Rigshospitalet, University of Copenhagen Copenhagen, Denmark; ^2^Department of Neuroanesthesia, Rigshospitalet, University of Copenhagen Copenhagen, Denmark; ^3^Department of Neuroscience and Pharmacology, University of Copenhagen Copenhagen, Denmark

**Keywords:** β_2_-adrenergic receptor gene, adrenergic β_2_-receptors, G16R, cardiac output, blood glucose, oxygenation glucose index

## Abstract

Cerebral non-oxidative carbohydrate consumption may be driven by a β_2_-adrenergic mechanism. This study tested whether the 46G > A (G16R) single nucleotide polymorphism of the β_2_-adrenergic receptor gene (*ADRB2*) influences the metabolic and cerebrovascular responses to administration of adrenaline. Forty healthy Caucasian men were included from a group of genotyped individuals. Cardio- and cerebrovascular variables at baseline and during a 60-min adrenaline infusion (0.06 μg kg^−1^ min^−1^) were measured by Model flow, near-infrared spectroscopy and transcranial Doppler sonography. Blood samples were obtained from an artery and a retrograde catheter in the right internal jugular vein. The *ADRB2* G16R variation had no effect on baseline arterial glucose, but during adrenaline infusion plasma glucose was up to 1.2 mM (CI_95_: 0.36–2.1, *P* < 0.026) higher in the Gly^16^ homozygotes compared with Arg^16^ homozygotes. The extrapolated steady-state levels of plasma glucose was 1.9 mM (CI_95_: 1.0 –2.9, *P*_NLME_ < 0.0026) higher in the Gly^16^ homozygotes compared with Arg^16^ homozygotes. There was no change in the cerebral oxygen glucose index and the oxygen carbohydrate index during adrenaline infusion and the two indexes were not affected by G16R polymorphism. No difference between genotype groups was found in cardiac output at baseline or during adrenaline infusion. The metabolic response of glucose during adrenergic stimulation with adrenaline is associated to the G16R polymorphism of *ADRB2*, although without effect on cerebral metabolism. The differences in adrenaline-induced blood glucose increase between genotypes suggest an elevated β_2_-adrenergic response in the Gly^16^ homozygotes with increased adrenaline-induced glycolysis compared to Arg^16^ homozygotes.

## Introduction

Cerebral energy metabolism at rest is provided almost exclusively by glucose and the molar ratio between the cerebral uptake of O_2_ to that of glucose (the O_2_-glucose index; OGI) is close to 6 (Quistorff et al., 2008). Adrenergic mechanisms influence cerebral energy metabolism (Bryan, 1990). Adrenaline increases the cerebral non-oxidative carbohydrate consumption (Seifert et al., 2009), presumably by a β_2_ adrenergic mechanism because propranolol, a combined β_1_- and β_2_-adrenergic receptor antagonist, attenuates cerebral carbohydrate uptake (Schmalbruch et al., 2002; Larsen et al., 2008), whereas metoprolol, a selective β_1_ adrenergic receptor antagonist, is without that effect (Dalsgaard et al., 2004). During maximal whole body exercise, the cerebral oxygen carbohydrate index (OCI; cerebral uptake of O_2_/(glucose + 1/2 lactate) decreases from a resting value of ~5.7 to reach a low value of 1.7 (Volianitis et al., 2008) that is associated with high levels of plasma catecholamine (Holmqvist et al., 1986; Nielsen, 2003).

The β_2_*-*adrenergic receptor is encoded by an intronless gene (*ADRB2*) located on chromosome 5 (5q31-32) that contains several single nucleotide polymorphisms (Leineweber et al., 2004). The non-synonymous 46 G > A (G16R) single nucleotide polymorphism leading to an amino acid substitution of Gly16Arg segregates with hypertension and asthma (Zaugg and Schaub, 2005; Sayers, 2013) and homozygote Gly^16^ subjects demonstrate a larger cardiac output (CO) both at rest and during exercise compared with homozygote Arg^16^ subjects (Snyder et al., 2006a; Rokamp et al., 2013). Differences in phenotype may arise from a higher receptor density in homozygote Gly^16^ subjects (Snyder et al., 2006b). However, differences in phenotype may also be a result of differences in sensitivity to β-agonists, as the Arg^16^ allele is associated with enhanced agonist-mediated desensitization (Dishy et al., 2001) and attenuated blood flow during infusion of a β-agonist in the brachial artery (Garovic et al., 2002). In contrast, Arg^16^ homozygotes had increased β_*2*_-receptor sensitivity after hypoglycemia whereas no effect was seen in Gly^16^ homozygotes (Schouwenberg et al., 2011). Also, the G16R polymorphism has been associated with insulin resistance (Masuo et al., 2005) and obesity (Daghestani et al., 2012), albeit with inconsistent results (Gjesing et al., 2009). Another polymorphisms in the β_*2*_*-*adrenergic receptor gene of functional importance is the 79C > G Q27E and in contrast to the Arg^16^ allele, the Glu27 allele is associated with increased agonist-mediated responsiveness in vasculature (Dishy et al., 2001). The role of haplotypes within *ADRB2* is, however, not known, but Rokamp et al. (2013) found no impact of haplotypes on cardiac output.

The adrenaline driven increase in cerebral non-oxidative carbohydrate consumption (Seifert et al., 2009), if mediated by a β_2_ adrenergic mechanism, could be influenced by genetic polymorphism in the β_2_*-*adrenergic receptor. We speculated that the difference in phenotype between Gly^16^ homozygotes and Arg^16^ homozygotes could mimic that Arg^16^ homozygotes was influenced by a β_2_ adrenergic receptor antagonist, leading to decreased cerebral carbohydrate uptake under adrenergic stress compared to Gly^16^ homozygotes.

No study describes the influence of genetic polymorphism in the β_*2*_-adrenergic system on brain metabolism. We aimed to investigate cardiovascular and cerebral metabolic effects of adrenergic stimulation in humans in relation to the G16R genotype. We hypothesized that cardiac output (CO) at rest and during adrenergic stimulation would be increased in Gly^16^ homozygotes and that the expected reduction in cerebral metabolic ratio during adrenergic stimulation would be more pronounced in Gly^16^ homozygotes, reflecting increased β_*2*_-adrenergic response compared to Arg^16^ homozygotes.

## Methods

Forty healthy non-smoking Caucasian male subjects (age: 26 ± 5 years; height: 184 ± 6 cm; body weight: 77 ± 8 kg; body mass index: 23 ± 2 kg/m^2^) were included in the study following verbal and written informed consent as approved by the Comittees on Biomedical Research Ethics of the Capital Region of Denmark, The Regional Committee A (H-4-2010-027) and the Danish Data Protection Agency (2011-41-6600). To obtain groups with similar age, height, and weight the subjects were recruited from a cohort of genotyped healthy subjects (Rokamp et al., 2013). All participants completed the entire study protocol. The genotype groups included 12 G16R heterozygotes, 12 Arg^16^ homozygotes, and 16 Gly^16^ homozygotes. Age, height, weight, and body mass index were similar in the three groups (Table 1).

**Table 1:** Cardiovascular variables during rest (baseline) according to the Gly16Arg polymorphism of the β_2_-adrenergic receptor gene (*n* = 40).

	**Baseline**			
	**GlyGly**	**GlyArg**	**ArgArg**	**P_ANOVA_**
N	*16*	*12*	*12*	*-*
Age (years)	25 ± 4	26 ± 6	26 ± 5	>1.0
Height (cm)	186 ± 6	183 ± 5	182 ± 7	>1.0
Weight (kg)	78 ± 8	78 ± 7	76 ± 9	>1.0
Body mass index (kg/m^2^)	23 ± 2	23 ± 2	23 ± 2	>1.0
SYS (mmHg)	130 ± 14	125 ± 9	125 ± 13	>1.0
DIA (mmHg)	67 ± 9	68 ± 5	65 ± 6	>1.0
MAP (mmHg)	87 ± 10	87 ± 8	85 ± 8	>1.0
HR(beat min^−1^)	63 ± 13	61 ± 10	57 ± 8	>1.0
SV (ml)	110 ± 7	106 ± 11	112 ± 5	>1.0
SVI (ml m^−2^)	55 ± 3	54 ± 4	58 ± 4	>1.0
CO (L min^−1^)	6.8 ± 1.2	6.4 ± 0.8	6.3 ± 1.0	>1.0
CI ((L min^−1^) m^−2^)	3.4 ± 0.8	3.2 ± 0.4	3.2 ± 0.5	>1.0
SVR (dyn s cm^−5^)	1,122 ± 277	1,112 ± 201	1,108 ± 224	>1.0
MCA Vmean	62 ± 10	60 ± 10	57 ± 11	>1.0
PI	0.8 ± 0.1	0.8 ± 0.2	0.8 ± 0.2	>1.0
ScO_2_ (%)	78 ± 8	77 ± 7	77 ± 4	>1.0
SmO_2_ (%)	82 ± 8	80 ± 10	81 ± 7	>1.0

*Values are mean ± SD. SYS, Systolic blood pressure; DIA, diastolic blood pressure; MAP, middle arterial pressure; HR, heart rate; SV, stroke volume; SVI, stroke volume index; CO, cardiac output; CI, cardiac index; SVR, systemic vascular resistance; MCA Vmean, mean flow velocity (a. cerebri media); PI, pulsatile index (a. cerebri media); ScO_2_, cerebral frontal lobe oxygenation; SmO_2_, muscle oxygenation. P_ANOVA_ values are corrected for multiple testing by the bonferroni method, therefore values above 1.0 appear*.

The subjects were studied after an overnight fast and strenuous exercise was not allowed 24 h prior to the study. Under local anesthesia (2% lidocaine), a catheter (Edwards Lifesciences, Irvine, CA) was inserted in the right internal jugular vein and advanced to its bulb using Seldinger technique. The position of the catheter was verified by a “water-fall-like” sound following infusion of saline and eventually by nociception related to the mastoid process and when so, the catheter was withdrawn about two millimeters. An arterial catheter (1.1 mm, 20 gauge) was inserted in the brachial artery of the non-dominant arm. For drug administration, a catheter (Cavafix MT134, Braun, Melsungen, Germany) was advanced to the subclavian vein through a cubital vein. Catheters were connected to a transducer (Edwards Life Sciences, Irvine, CA) positioned at heart level (5 cm below sternum) and attached to a monitor (Dialogue-2000 IBC-Danica Electronic, Denmark) for determination of mean arterial pressure (MAP) and heart rate (HR). Stroke volume (SV), CO and systemic vascular resistance (SVR) were derived by pulse contour analysis technology (BeatScope; Finapress Medical System BV, Amsterdam, Netherlands) adjusting for weight, height, age, and gender. Data were analog-digital converted and sampled at 100 Hz (Powerlab, ADInstruments, Colorado Springs, CO, USA).

Near infrared spectroscopy (INVOS-5100c, Covidien, Mansfield, MA, USA) was used to assess frontal lobe (S_c_O_2_) and muscle oxygenation (S_m_O_2_). The INVOS-5100c uses an emitter-detector distance of 3 and 4 cm and infrared light at 730 and 808 nm to avoid influence from cutaneous blood flow. One optode were applied above the supraorbital edge to assess S_c_O_2_ and a second optode was placed on the middle part of the thigh for assessment of S_m_O_2_. Transcranial Doppler sonography (2 MHz probe, Multi-Dop, DWL, Singen, Germany) determined velocity in the middle cerebral artery (MCAv_mean_) from the temporal ultrasound window. The best signal-to-noise ratio was obtained at a depth of 44–56 mm with the Doppler probe secured by a headband or while handheld.

### Protocol

Following catheterization the subjects' rested supine for 30 min. Adrenaline was prepared in 100 ml isotone saline solution according to weight and infused for 60 min at 0.06 μg kg^−1^ min^−1^. After termination of the infusion, the subjects were observed for another 30 min. Simultaneous arterial and venous blood samples were obtained in pre-heparinized syringes (QS50, Radiometer, Copenhagen, Denmark) and immediately purged of any atmospheric content followed by analysis using an ABL 725 (Radiometer). Blood sampling and cardiovascular variables were obtained at rest and at 2.5 min intervals during the initial 10 min of the infusion and thereafter at 10 min intervals until the infusion was terminated.

### Purification of DNA and genotyping

DNA was purified from 200 μl frozen blood samples by the magnetic bead based MagneSil® Blood Genomic, Max Yeld System (Promega, Madison WI, USA). Genotyping was performed using TaqMan assay with the following rs and AB number: rs1042713, c___2084764_20. The assay was analyzed using real-time polymerase chain reaction by an Applied Biosystem 7,500 Fast Real Time polymerase chain reaction device according to the manufacturer's instruction (Applied Biosystem, Lincoln, CA, USA).

### Calculations

The OCI and the ratio taking only glucose into account (OGI, O_2_/glucose; Fox et al., 1988) were calculated and both ratios were considered independent of changes in cerebral blood flow (CBF) (Dalsgaard, 2006). Although pyruvate is a viable carbohydrate source in fueling cerebral activity, pyruvate was omitted in the analysis based on the assumption that its uptake by the brain is at least an order of magnitude smaller than that of lactate (Rasmussen et al., 1985).

The cumulated cerebral uptake of glucose, lactate and O_2_ was calculated from the arterial and internal jugular venous concentrations assuming a resting CBF of 700 ml min^−1^ (Jørgensen et al., 1992), adjusted according to changes in MCAv_mean_ (Quistorff et al., 2008): Σ*t*_(0)_/*t*_(n)_ substrate uptake = {(*t*_1_ - *t*_0_) × [arterial– venous difference substrate_1_] × CBF_1_ + (*t*_2_ − *t*_1_) × [arterial– venous difference substrate_2_] × CBF_2_ + (*t*_*n*_ − *t*_*n*__−1_) × [arterial– venous difference substrate_*n*_] × CBF_*n*_}.

### Statistics

Statistically analysis was performed using R version 3.0.3 with add-on packages: “nlme” (Pinheiro, Bates, DebRoy, Sarkar, and R Core Team, version 3.1-113), “ggplot2,” “grid,” and “reshape 2” attached. The alpha-level was set to 5%. Baseline data were analyzed with standard parametric models [Analysis of variance (ANOVA) and/or *t*-test] or non-parametric tests (Kruskal-Wallis) if residual analysis revealed non-normal distributions. For repeated measurements of CO, arterial-glucose and arterial-lactate, a non-linear mixed effects model (NLME) was used to take into account within subject correlated data. The proposed dose-response relationship is based on a standard first-order pharmacokinetic model following the formula:

$$f(t)=\beta \cdot (1-e^{\text{-}\gamma \;\cdot \;t})+\alpha$$

Each individual was allowed (i.e., random effect of) his own values of intercept (α), steady-state (β) and rate of increase (γ) parameters. Briefly, α models the individual's baseline value, β is the estimated value approached asymptotically as time increases (in practice around 60 min), and γ determines the steepness of the initial slope. A genotype effect on each of the parameters α, β, and γ was tested with the “nlme” package. Linear mixed effects models (LME) were constructed in cases where the kinetic model (Equation 1) could not be fitted using the non-linear mixed effects function in R. In each group the change from baseline was analyzed based on the mixed model with the main effects of group, time, and interaction. Models were fit using maximum-likelihood. Assumptions of normality of error-distribution were assessed with residual plots. In addition, models were validated by influence analysis to verify that no single measurement or individual could change conclusions. Mean values with SD are reported unless otherwise indicated. The bonferroni method was used to correct for multiple comparisons. Thus *P*-values was multiplied with the number of tested variables (*n* = 26) and therefor *P* > 1.0 can appear in the text, *P*-values lower than 0.05 were regarded as statistically significant.

## Results

### Cardiovascular variables

**Figure 1:**
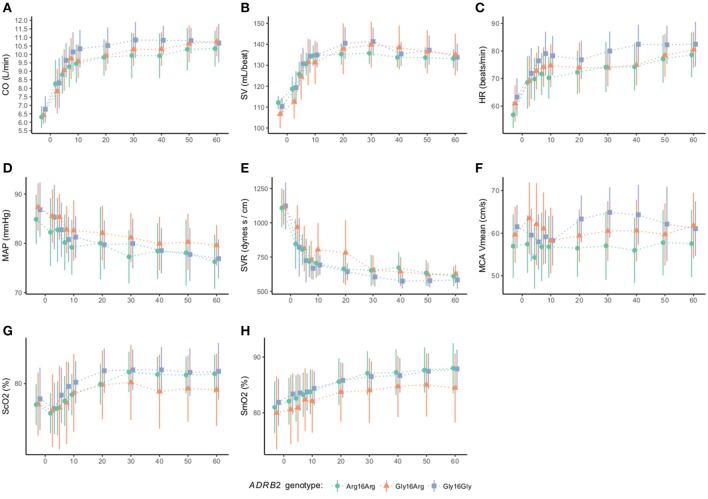
Cardiovascular variables according to the β_2_-adrenergic receptor gene G16R polymorphism at baseline and during adrenaline infusion (*n* = 40). The mean and SD of each genotype group is presented to each time point. Baseline measurements are followed by adrenaline infusion that is initiated at time 0. Cardiac output (CO), heart rate (HR), stroke volume (SV), middle arterial pressure (MAP), systemic vascular resistance (SVR), cerebral frontal lobe oxygenation (S_c_O_2_), muscle oxygenation (S_m_O_2_), and mean middle cerebral artery velocity (MCAv_mean_). There were no significant differences between genotypes in any of the cardiovascular variables. Adrenaline increased CO (*P* < 0.0026) **(A)**, SV (*P* < 0.0026) **(B)**, HR (*P* < 0.0026) **(C)**, ScO_2_ (*P* < 0.0026) **(G)**, S_m_O_2_ (*P* < 0.0026) **(H)** compared to baseline, but decreased MAP (*P* < 0.0026) **(D)** and SVR (*P* < 0.0026) **(E)**, while MCAv_mean_ did not change significantly from baseline (*P* > 1.0) **(F)**. Following termination of the adrenaline infusion cardiovascular variables approached the baseline levels.

Cardiovascular variables are presented in Figure 1. Cardiovascular variables showed no significant differences between genotypes at baseline (Table 1) or during adrenaline infusion (Figure 1).

### Metabolic variables

**Figure 2:**
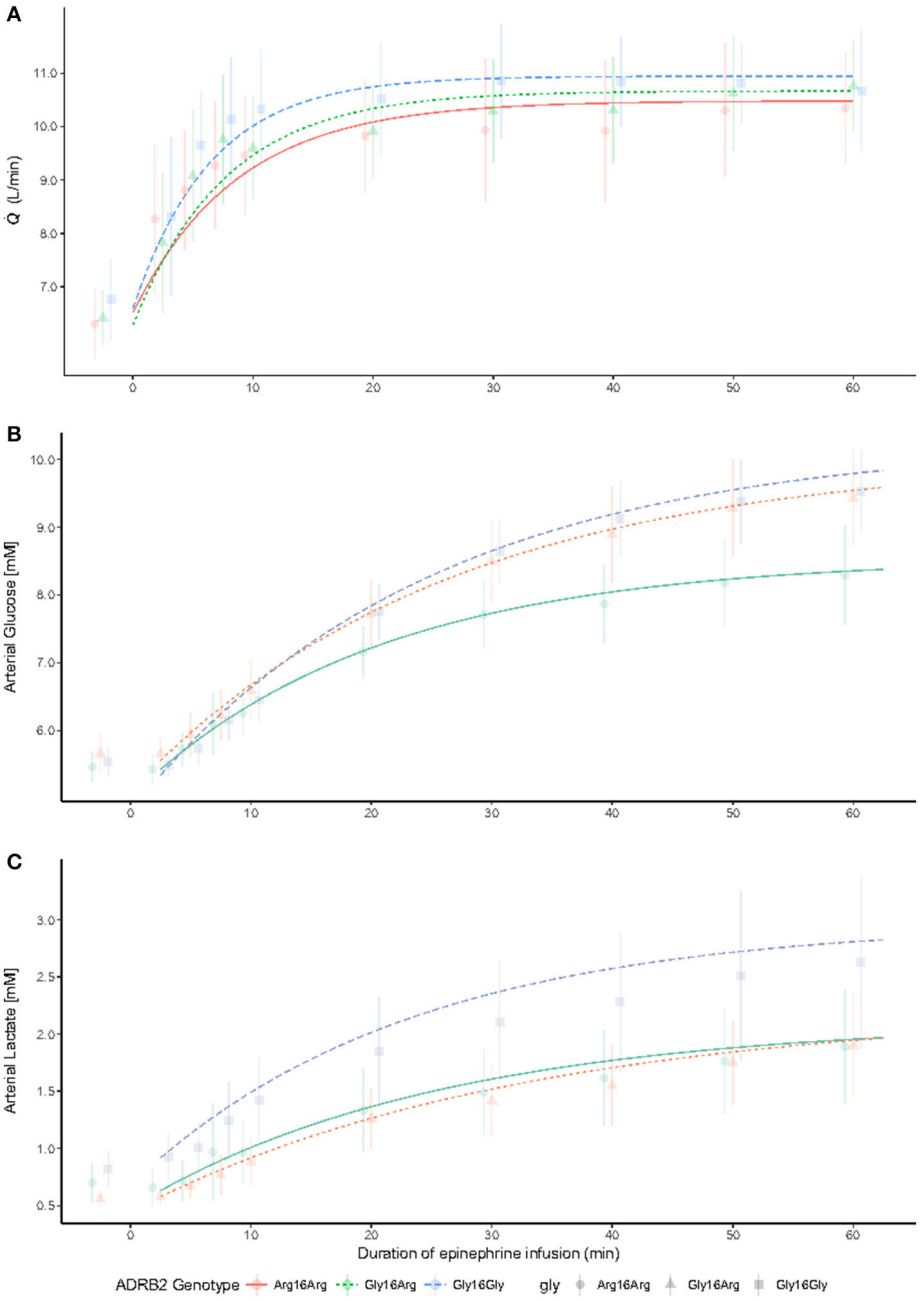
Cardiac output (CO), arterial glucose and arterial lactate according to the β_2_-adrenergic receptor gene G16R polymorphism at baseline and during adrenaline infusion (*n* = 40). Baseline measurements are followed by adrenaline infusion that is initiated at time 0. The figure shows the non-linear mixed effects model (Equation 1), with an underlay were the mean and SD of each genotype group is presented to each time point. There were no significant differences between genotypes in CO (*P*_ANOVA_ > 1.0) **(A)**. Arterial glucose **(B)** was 1.2 mM (CI_95_: 0.36−2.1, *P* < 0.026) higher in the Gly^16^ homozygotes compared with Arg^16^ homozygotes after 60 min of adrenaline infusion. At the extrapolated steady-state level (not shown on the figure) the Gly^16^ homozygotes had an arterial glucose that was 1.9 mM (CI_95_: 1.0–2.9, *P*_NLME_ < 0.0026) higher than in the Arg^16^ homozygotes. There were no significant differences between genotypes in arterial lactate (*P*_ANOVA_ = 0.78) (**C**).

**Figure 3:**
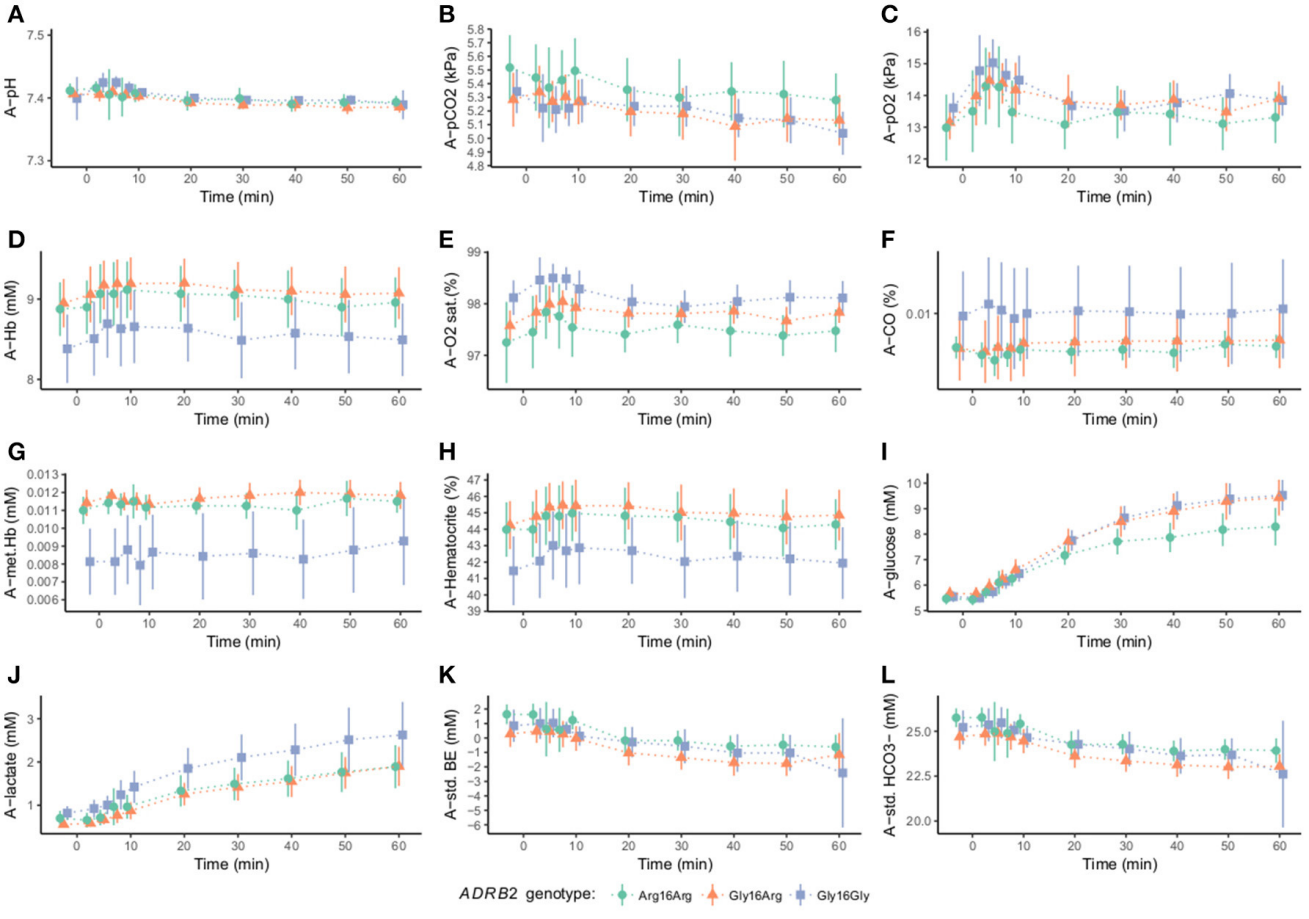
Arterial blood gas variables according to the β_2_-adrenergic receptor gene G16R polymorphism at baseline and during adrenaline infusion (*n* = 40). The mean and SD of each genotype group is presented to each time point. Baseline measurements are followed by adrenaline infusion that is initiated at time 0. Arterial pH (pH), arterial CO_2_ tension (pCO_2_), arterial O_2_ tension (pO_2_), arterial hemoglobin (Hb), arterial oxygen saturation (O_2_ sat.), arterial fraction of carboxyhemoglobin (COHb), arterial methemoglobin (metHb), arterial standard base excess (std. BE), and arterial standard hydrogen carbonate (HCO_3_−). Following baseline arterial blood gas variables for did not differ between genotypes, pH (*P*_ANOVA_ > 1.0) **(A)**, pCO_2_ (*P*_ANOVA_ > 1.0) **(B)**, pO_2_ (*P*_ANOVA_ > 1.0) **(C)**, Hb (*P*_ANOVA_ > 1.0) **(D)**, O_2_ sat (*P*_ANOVA_ < 0.68) **(E)**, Hb (*P*_ANOVA_ > 1.0) **(F)**, hematocrite (*P*_ANOVA_ > 1.0) **(H)**, Glucose (*P*_ANOVA_ > 1.0) **(I)**, lactate (*P*_ANOVA_ = 0.78) **(J)**, std. BE (*P*_ANOVA_ > 1.0) **(K)**, std HCO_3_− (*P*_ANOVA_ > 1.0) **(L)**. Except for glucose none of these variables was associated with a genotype specific difference during adrenaline infusion. In metHb a genotype specific difference was found both at baseline and during adrenaline infusion (*P* = 0.0234) **(G)**. Adrenaline infusion however, did not change the levels of methemoglobin, as compared to baseline (*P* < 0.098).

Distributions of metabolic variables are presented in Figures 2,3. Baseline arterial glucose was 5.6 ± 0.4 mM with no significant differences between genotypes (*P*_ANOVA_ > 1.0). During adrenaline infusion (after 60 min of infusion) plasma glucose was up to 1.2 mM (CI_95_: 0.36-2.1, *P* < 0.026) higher in the Gly^16^ homozygotes compared with Arg^16^ homozygotes. Fitting the non-linear mixed effects model (Equation 1), there was an effect of the G16R polymorphism on the extrapolated steady-state level (β) (*P*_NLME_ < 0.0026), but not on the intercept (α), or rate of increase (γ). At the extrapolated steady-state level (not shown on the figure) the Gly^16^ homozygotes had an arterial glucose that was 1.9 mM (CI_95_: 1.0–2.9, *P*_NLME_ < 0.0026) higher than in the Arg^16^ homozygotes. There was no significant difference in arterial glucose during adrenaline infusion between the G16R heterozygotesand Arg^16^ homozygotes (*P*_NLME_ = 0.16).

Baseline arterial lactate was 0.7 ± 0.3 mM without differences between genotypes after correction for multiple testing (*P*_ANOVA_ = 0.78). The non-linear mixed effects model showed no significant effect of the G16R polymorphism on the baseline (intercept) parameter α (*P*_NLME_ > 1.0), the steady state parameter β (*P*_NLME_ > 1.0), or on the rate of increase γ (*P*_NLME_ > 1.0) (Figure 2C).

In methemoglobin a genotype specific difference was found both at baseline and during adrenaline infusion (*P* = 0.0234) (Figure 3G). Adrenaline infusion however, did not change the levels of methemoglobin, as compared to baseline (*P* < 0.098).

All other baseline arterial blood gas variables did not differ between genotypes and in none of these variables a genotype specific difference during adrenaline infusion was found. All metabolic variables changed during adrenaline infusion as shown in Figure 3.

### Whole brain metabolism

**Figure 4:**
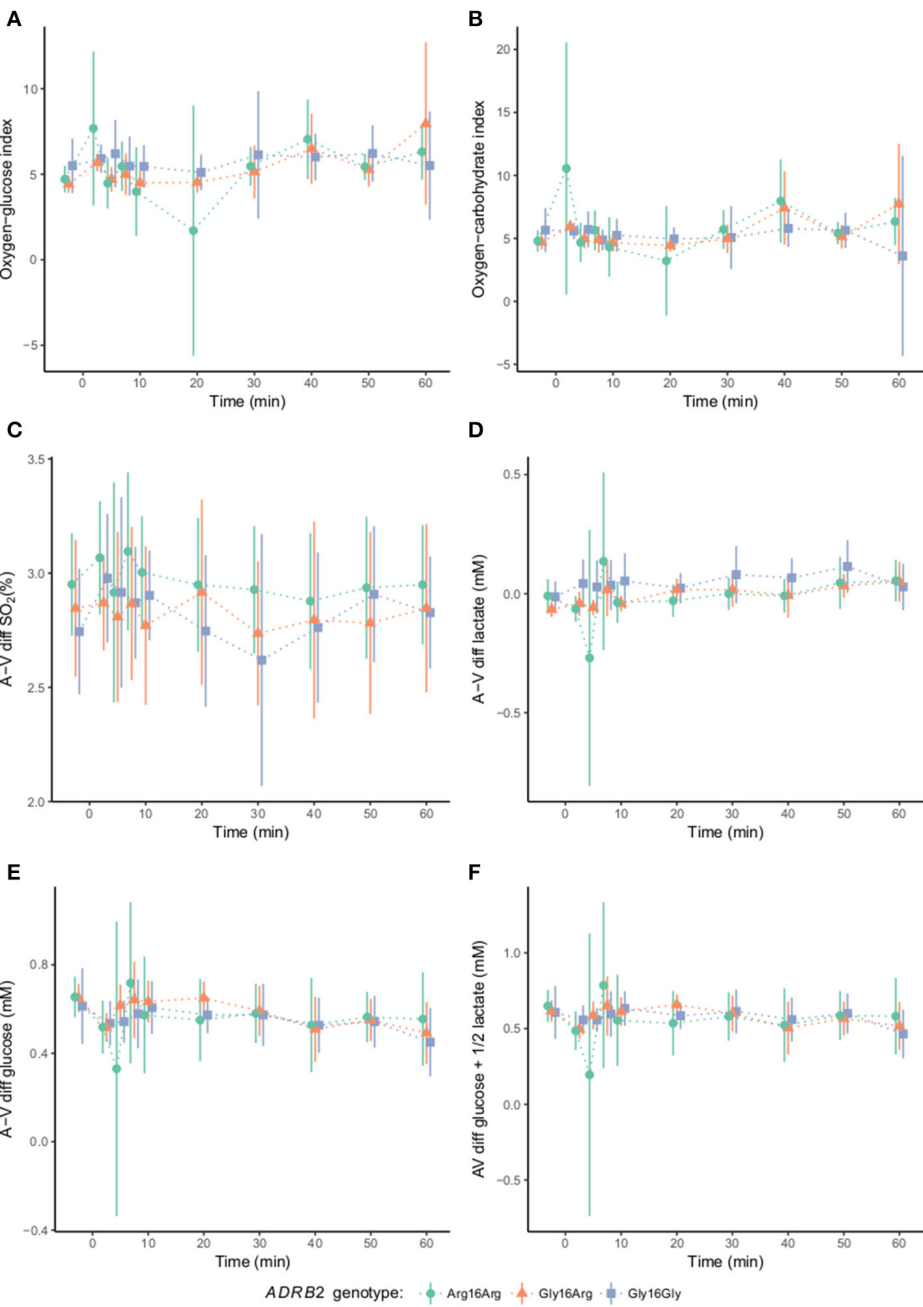
Cerebrovascular variables according to the β_2_-adrenergic receptor gene G16R polymorphism at baseline and during adrenaline infusion (*n* = 40). Baseline measurements are followed by adrenaline infusion that is initiated at time 0. Oxygen-glucose index (OGI), oxygen-carbohydrate index (OCI), arterial—venous difference in oxygen saturation (A-V diff SO_2_), arterial—venous difference in lactate (A-V diff lactate), arterial—venous difference in glucose (A-V diff glucose), arterial—venous difference in glucose + ½ lactate (A-V diff glucose + ½ lactate). There were no significant changes in the brain metabolic indices at baseline or at adrenaline infusion, oxygen-glucose index (*P*_LME_ = 0.26) **(A)** oxygen carbohydrate index (*P*_LME_ > 1.0) **(B)**, A-V diff SO_2_ (*P*_LME_ = 0.52) **(C)**, A-V diff lactate (*P*_LME_ = 0.16) **(D)**, A-V diff glucose (*P*_LME_ = 0.78) **(E)** and A-V diff glucose + ½ lactate (*P*_LME_ > 1.0). There were no significant differences between the genotype groups in any of the brain metabolic indices.

The non-linear model (Equation 1) did not fit the brain metabolic indices. Results from linear mixed effects models including duration of adrenaline infusion as predictor of the investigated brain metabolic indices are reported in Figure 4. With the homozygote Gly^16^ polymorphism as a predictor of both slope (interaction) and intercept (additive effect), there was no significant effect of genotype in any of the brain metabolic indices.

## Discussion

In contrast to our hypothesis, the cerebral uptake of glucose was not different among the genotype groups. Seifert et al. (2009) suggest that adrenaline is responsible for the increase in non-oxidative cerebral carbohydrate consumption. Comparing this study with the work by Seifert et al. (2009), the adrenaline infusion rate was lower (0.06 vs. 0.08 μg kg^−1^ min^−1^), and our subjects increased their heart rate to below 80 bpm compared to 90 bpm, suggesting a difference in adrenergic stimulation. Apart from the study of Seifert et al. (2009), the hypothesis that adrenaline should stimulate the cerebral uptake of glucose and lactate, generates from studies on the effects of exercise in rats (Schmalbruch et al., 2002) and humans (Dalsgaard et al., 2004; Larsen et al., 2008; Volianitis et al., 2008) were it is likely that the adrenergic stimulation was increased compared to that in the present study.

Snyder et al. (2006a) and Rokamp et al. (2013) found baseline CO increased in Gly^16^ homozygotes compared with Arg^16^ homozygotes. We found the same effect size (~0.5 L min^−1^) although the difference was not statistically significant. This study differs from the previous studies by the population consisting of young males compared to mixed groups (Snyder et al., 2006a and Rokamp et al., 2013) of variable age (Rokamp et al., 2013), but a smaller population size (*n* = 40 compared to *n* = 72 (Snyder et al., 2006a) and *n* = 140 (Rokamp et al., 2013), respectively) and while Snyder et al. (2006a) used the open-circuit acetylene uptake method, we used the Model-flow method (Bogert and van Lieshout, 2005). Model-flow seems to underestimate the increase in CO during heat stress (Shibasaki et al., 2011), but has been successfully validated against a thermodilution estimate in healthy subject during orthostatic stress (Harms et al., 1999) and in patients with septic shock (Jellema et al., 1999), during liver transplantation and cardiac surgery (Jansen et al., 2001; Nissen et al., 2009).

The systemic increase in glucose following adrenaline stimulation is expected from increased endogen glucose production and a sustained inhibitory effect on glucose clearance (Rizza et al., 1980a). The β-adrenoceptor subtype that mediates catecholamine-induced systemic hyperglycemia is proposed to be of the β_*2*_-subtype (Kuo et al., 1977), and the adrenaline derived decrease in glucose clearance is predominantly by a β-adrenergic mechanism (Rizza et al., 1980b). This complies with the finding that the elevation in arterial glucose is associated with the *ADRB2* Gly^16^ polymorphism and may be explained by an elevated β_2_-adrenergic response in the Gly^16^ homozygotes. The difference in metabolic response according to genotype may arise from a difference in number of receptors, i.e., Snyder et al. (2006b) found an increased density of β_*2*_-receptors on lymphocytes from Gly^16^ homozygotes compared with Arg^16^ homozygotes.

In patients with longstanding type 1 diabetes, blunting of the glucagon response comes along with the disappearing endogenous insulin production (Cryer, 2008), rendering the patients increasingly dependent upon epinephrine as protection against hypoglycemia. As a result of recurrent hypoglycemia and/or long duration of diabetes, also the epinephrine response to hypoglycemia becomes blunted, leading to an increased risk of severe hypoglycemia (Høi-Hansen et al., 2010). In addition to the failing catecholamine response to hypoglycemia with impaired hypoglycemia awareness, a reduced β_2_-adrenergic sensitivity has been reported in some (Korytkowski et al., 1998; Fritsche et al., 2001), but not all studies (De Galan et al., 2006). In accordance, treatment with non-specific β-blockers with effect on the β_2_-receptor is associated with reduced endogenous glucose production to adrenaline infusion (Shamoon and Sherwin, 1984), impaired recovery from hypoglycemia (Lager, 1983; Popp et al., 1984), and probably an increased risk of severe hypoglycemia in type 1 diabetes. The difference between genotypes in systemic adrenergic glucose response is novel and we speculate that it may be of clinical importance in patients with type 1 diabetes. The glucose response in this study was under conditions with baseline normoglycemia and cannot be compared to the conditions during hypoglycemia. Further studies are needed to uncover the genetic impact on glucose mobilization during hypoglycemia.

## Limitations

The limitations of the study include not monitoring plasma insulin. There were no differences in arterial glucose level at baseline, but the Gly^16^ allele has been associated with increased insulin levels (Ikarashi et al., 2004) and plasma insulin increase initially in response to adrenaline (Sherwin and Saccà, 1984).

Plasma catecholamine levels are not monitored in this study, but earlier there have been found no difference between genotypes in catecholamine levels during rest or exercise (Snyder et al., 2006a).

The steady state of plasma glucose is not reached during 60 min of adrenaline infusion, and differences in plasma glucose is therefore lower than would be expected if time of adrenaline infusion had been extended.

## Summary and conclusion

An association was found between the G16G genotype and adrenaline induced increase in arterial glucose with no difference at baseline. We found no other relevant differences between genotypes in any other measured cardiovascular or cerebral variable at baseline or during adrenaline infusion. In conclusion, the metabolic response of glucose during adrenergic stimulation with adrenaline is associated to the G16R polymorphism of *ADRB2*, although without effect on cerebral metabolism.

## Author contributions

KR participated in study design, collected the data, performed data analysis and wrote the first draft of the paper. JS, MZ, LP, and RN collected data, performed data analysis and contributed to preparation of the paper. PR performed data analysis and contributed to preparation of the paper. NS, NO, and HN participated in study design, performed data analysis, and contributed to preparation of the paper. All authors approved the manuscript and agree to be accountable for all aspects of the work in ensuring that questions related to the accuracy or integrity of any part of the work are appropriately investigated and resolved.

### Conflict of interest statement

The authors declare that the research was conducted in the absence of any commercial or financial relationships that could be construed as a potential conflict of interest.

## Abbreviations

*ADRB2*: β_2_-adrenergic receptor gene
ANOVA: analysis of variance
CBF: cerebral blood flow
CO: cardiac output
HR: heart rate
LME: linear mixed effects models
MAP: mean arterial pressure
MCAv_mean_: velocity in the middle cerebral artery
NLME: non-linear mixed effects model
OCI: the cerebral oxygen carbohydrate index
OGI: the cerebral O_2_-glucose index
SV: stroke volume
SVR: systemic vascular resistance
S_c_O_2_: frontal lobe oxygenation
S_m_O_2_: muscle oxygenation.
